# Cardiovascular disease related death among patients with esophagus cancer: A population-based competing risk analysis

**DOI:** 10.3389/fonc.2022.976711

**Published:** 2022-09-15

**Authors:** Yang Xia, Min Lin, Jin Huang, Li Fan

**Affiliations:** Department of Gastroenterology, The Affliated Changzhou No.2 People’s Hospital of Nanjing Medical University, Changzhou, China

**Keywords:** esophagus cancer, cause of death, cardiovascular disease, surveillance, epidemiology, and end result, standardized mortality rates

## Abstract

The proportion of non-cancer death in patients with esophagus cancer (EC) still increasing, especially cardiovascular disease (CVD) related death. The aim of this study was assess non-cancer causes of death and identified independent risk factors of CVD related death in EC patients. Patients diagnosed with EC were extracted from the Surveillance, Epidemiology, and End Result database (SEER) database for analysis. Standardized mortality rates (SMRs) for non-EC deaths were calculated, the risk of death were assessed and compared with US general population. Multivariate competitive risk analysis were performed to select independent risk factors for death from CVD in EC patients. A total of 43739 EC patients were enrolled and 35139 died during follow-up, of which 4248 died from non-cancer cause of death. The risk of non-cancer death in EC patients was 2.27-fold higher than in the general population (SMR=2.27; 95% CI, 2.20-2.34). CVD were the most important cause of non-cancer death in EC patients, accounting for 43.4% of non-cancer of deaths. Compare with the general population, EC patients have higher risk of death from disease of heart (SMR, 2.24; 95% CI, 2.13-2.35), pneumonia and influenza (SMR, 2.92; 95% CI, 2.50-3.39), septicemia (SMR, 5.01; 95% CI, 4.30-5.79), along with other causes. Patients with advanced age and patients who received radiotherapy has higher risk of death caused by CVD, patients with female sex, poor differentiated and undifferentiated, regional and distant stage, married, diagnosed between 2010-2016 has lower risk of CVD related death, compared with patients without any treatment measures, patients received chemotherapy alone has lower risk of death from CVD. Non-cancer cause of death has become an important cause of death in EC patients. Improving public awareness of the major risk factors for non-cancer death is beneficial to the prevention and treatment of malignant tumors.

## Introduction

Esophagus cancer (EC) is seventh common cancers and the sixth leading cause of cancer death worldwide, with 604100 new cases and 544076 deaths in 2020, respectively (1).

With recent advances in cancer prevention, diagnosis and treatment measures, the number of cancer survivors continues to rise, these patients are not only at risk for recurrence, but may also have a variety of disease states, result the risk of non-cancer death becoming a huge threat to the health of cancer survivors (2, 3). Due to the improvement of treatments and the popularity of endoscopic screening of the upper gastrointestinal tract has led to more EC patients being diagnosed at an earlier stage, the 5-years overall survival rates of EC patients has improved in recent years (4–9). This part of EC patients may live long enough after the initial diagnosis, resulting in non-cancer-related comorbidities that may have a dramatic impact on overall survival. Recent years, the proportion of non-cancer death in EC patients were increasing, especially cardiovascular disease (CVD) related death (2, 10). Therefore, understanding the proportion and risk factor of different causes of death after EC diagnosis can be helpful in EC patient’s follow up and management. This study was designed to comprehensively assess non-cancer causes of death and identified independent predictors of CVD related death in EC patients, to provide a constructive basis for follow-up management of EC patients and optimize their quality of life.

## Materials and methods

### Data source and study population

This retrospective cohort study used data from the Surveillance, Epidemiology, and End Results (SEER) database, it’s a population-based cancer registry sponsored by the National Cancer Institute which includes morbidity, survival, and mortality data and covering approximately 27.8% of the total US population. Because of its population-based program design, data selected in SEER program can be used for comparison with the general population, and therefore can be used to estimate cancer incidence, mortality, and survival (11). We used SEER*stat software to extract primary EC patients diagnosed between 2000-2016, patients diagnosed only at autopsy or on death certificates were not included. Public information provided by the SEER program does not require ethical approval.

### Variable

**Figure 1:**
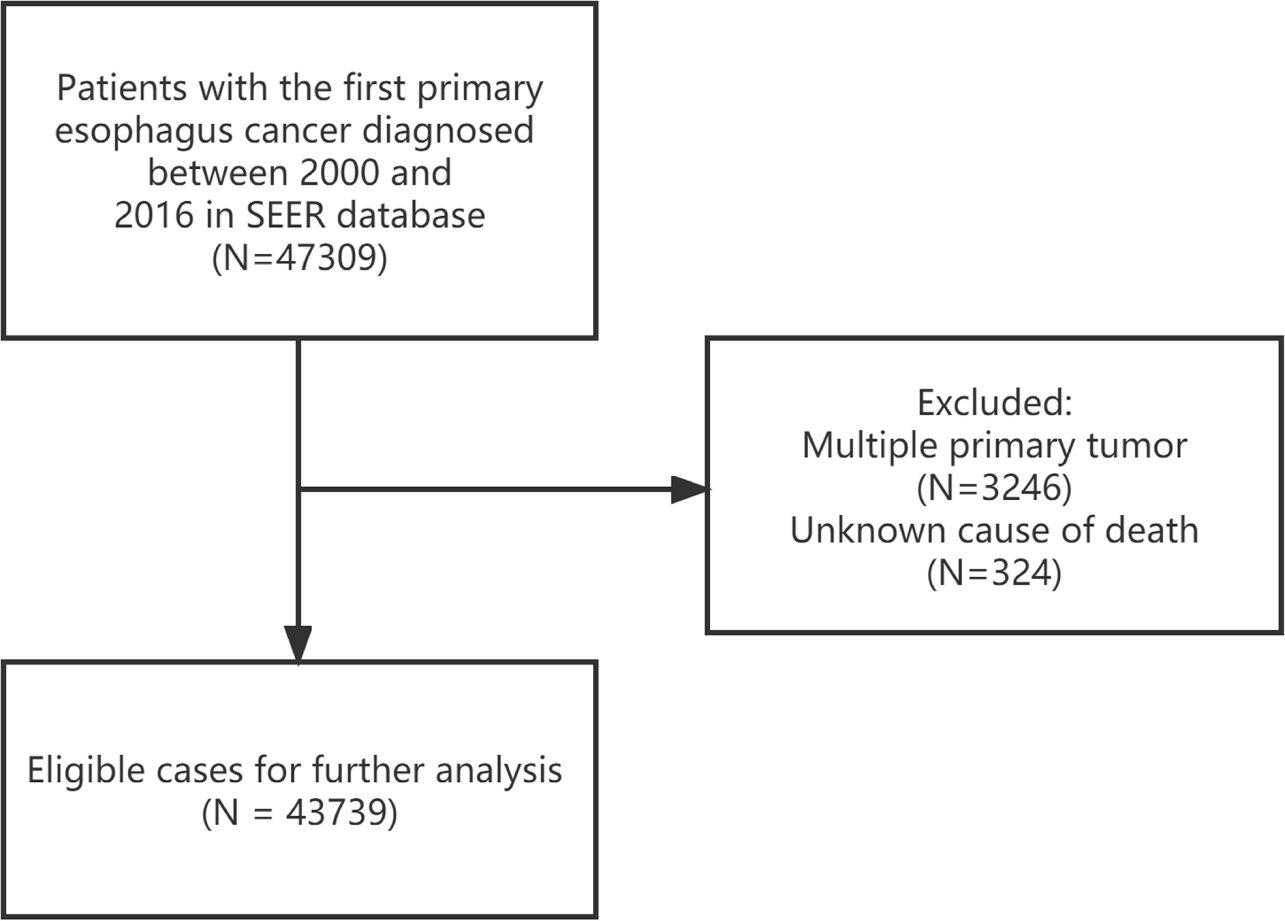
Inclusion and exclusion flowchart.

EC Patients (Site recode International Classification of Diseases for Oncology-3 (ICD-O-3)/WHO 2008: Esophagus) with malignant behavior between 2000-2016 were extracted from SEER database. The exclusion criteria were as follow (1): Multiple primary tumor; (2) Unknown cause of death; the specific process is shown in **Figure 1**. Patient’s detailed information were extracted including age at diagnosis, sex, race, diagnosis year, insurance status, marital status, grade, primary site, SEER summary stage, surgery, radiotherapy and chemotherapy. Well-differentiated and moderately differentiated tumors were defined as grade I + II, poorly differentiated and undifferentiated tumors were defined as grade III + IV.

### Classification of cause of death

In the SEER database, the cause of death classification is coded according to the International Classification of Diseases, Version 9 (ICD-9) and ICD-10 used by the registry, information about cause of death categories in the general population originate in the Wide-ranging Online Data for Epidemiologic Research tool from the Centers for Disease Control and Prevention and which can available through the SEER*stat software (12). SEER database through systematic and standard data collection procedure to ensure the accuracy of cause of death determinations, thereby mitigating the possible impacts of potential biases on study findings (13). All cause of death was referring to the total number of deaths during the follow-up period, non-cancer death included seven categories: cardiovascular disease, infectious disease, respiratory disease, kidney disease, gastrointestinal disease, external injury, and other causes of death. CVD included disease of heart, hypertension without heart disease, cerebrovascular diseases, aortic aneurysm and dissection, atherosclerosis and other diseases of arteries, arterioles, capillaries (14).

### Outcome

We examined non-cancer causes of death in EC patients, mortality events were classified according to the time interval from diagnosis to death, these intervals contained: <1 year, 1-5 years, 5-10 years and >10 years. The relative risk of non-cancer death among EC patients was compared with that of US general population and expressed as standardized mortality rates (SMRs), which was defined as the radio of observed number divide expected number, ”Observed” was defined as the actual death number of a specific cause in a specific interval, and “Expected” was defined as the expected number of deaths from the same cause in a demographically similar population (regarding sex, race, and age) within the same interval (15).

### Statistic analysis

SMR and 95% confidence intervals (95%CI) were calculated in different interval after diagnosis (<1 year, 1-5 years, 5-10 years and >10 years), we used the Poisson exact method to compute the 95% CI for SMR. The hazard ratios (HRs) and 95% CI were used to estimate the associations between patient characteristics and CVD related death in EC patients through multivariate competing risk analysis. Death from CVD were defined as events of primary concern, while competitive events were defined as deaths from other causes. All tests were double-sided, P < 0.05 were considered statistically significant. Data analysis was performed by SEER*stat (version 8.4.0) and R software (version 4.0.0).

## Results

### Baseline characteristics

After above exclusion criteria, 43739 EC patients were extracted from the SEER database for further analysis. Most patients died within 5 years after diagnosis (94.6%). The majority of patients were male (78.7%), white patients accounted for 83.7% of the total. Most included patients were poor differentiation and undifferentiation (41.8%), married (55.3%), insured (48.5%), 50-70 years old (56.7%) and were located in the lower third of the esophagus (60.6%). Most esophageal malignancies were found in regional stage (33.6%) and distant stage (34.2%), while only a minority of patients were diagnosed in the localized stage (21.5%). The rate of surgery, radiotherapy and chemotherapy in our cohort were 28%, 59.4% and 64.5%, respectively. Some patients received multiple treatment measures, as shown in **Table 1** for specific information.

**Table 1:** Baseline characteristics of EC patients.

Characteristic		All diagnosis cases (n)
All patients with EC		43739
Age	<50	3530
	50-70	24799
	>70	15410
Race	White	36608
	Black	4924
	Other	2207
Sex	Female	9309
	Male	34430
Grade	I+II	16452
	III+IV	18270
	Unknown	9017
Stage	Localized	9424
	Regional	14699
	Distant	14965
	Unknown	4651
Primary site	Upper esophagus	2868
	Mid- esophagus	8223
	Lower esophagus	26504
	Overlap	1847
	Unknown	4297
Diagnosis year	2000-2009	25257
	2010-2016	18482
Marital status	Married	24204
	Single	17351
	Unknown	2184
Insured status	Insured	21208
	Medicaid	3443
	Uninsured	856
	Unknown	18232
Surgery	No+Unknown	31492
	Yes	12247
Radiotherapy	No+Unknown	17753
	Yes	25986
Chemotherapy	No+Unknown	15539
	Yes	28200
Treatment measures	No+Unknown	7407
	Surgery only	4332
	Radiotherapy only	3494
	Chemotherapy only	5179
	Surgery+ Radiotherapy	306
	Surgery+ Chemotherapy	835
	Radiotherapy+ Chemotherapy	15412
	Surgery+ Radiotherapy+ Chemotherapy	6774

### Non-cancer causes of death

A total of 35,193 patients died during follow-up, of which 4248 (12.1%) died from non-cancer causes, including CVD (1843, 43.4%), other cause of death (1109, 26.1%), infectious diseases (451, 10.6%), respiratory diseases (439, 10.3%), external injuries (228, 5.4%), renal diseases (91, 2.1%) and gastrointestinal diseases (87, 2.0%). Throughout the follow-up period, patients had a higher risk of death from disease of heart (SMR, 2.24; 95% CI, 2.13-2.35), cerebrovascular diseases (SMR, 1.56; 95% CI, 1.35-1.78), pneumonia and influenza (SMR, 2.92; 95% CI, 2.50-3.39), septicemia (SMR, 5.01; 95% CI, 4.30-5.79), COPD (SMR, 2.75; 95% CI, 2.50-3.02), suicide and self-inflicted injury (SMR, 4.08; 95% CI, 3.24-5.06) and nephritis, nephrotic syndrome and nephrosis (SMR, 1.79; 95% CI, 1.44-2.20) compared with US general population, along with other cause (**Table 2**).

**Table 2:** Standardized-mortality ratios for each cause of death following esophagus cancer diagnosis.

Cause of death	< 1 year		1-5 years		5-10 years		> 10 years		Total	
	Observed	SMR(95%CI)	Observed	SMR(95%CI)	Observed	SMR(95%CI)	Observed	SMR(95%CI)	Observed	SMR(95%CI)
**ALL cause of death**	20335	29.20 (28.80-29.61)	12962	11.67 (11.47-11.87)	1481	2.85 (2.70-2.99)	395	2.10 (1.89-2.31)	35193	13.99 (13.84-14.13)
**Non-cancer of death**	1769	3.42 (3.27-3.59)	1599	1.95 (1.86-2.05)	645	1.65 (1.53-1.79)	235	1.62 (1.42-1.84)	4248	2.27 (2.20-2.34)
**Cardiovascular diseases**	756	3.04 (2.83-3.27)	689	1.79 (1.66-1.93)	286	1.62 (1.43-1.81)	112	1.75 (1.44-2.10)	1843	2.11 (2.01-2.20)
Diseases of heart	621	3.22 (2.97-3.48)	573	1.91 (1.75-2.07)	237	1.72 (1.51-1.96)	93	1.87 (1.51-2.29)	1524	2.24 (2.13-2.35)
Hypertension without heart disease	19	2.76 (1.66-4.31)	17	1.53 (0.89-2.45)	6	1.05 (0.39-2.29)	3	1.35 (0.28-3.95)	45	1.74 (1.27-2.32)
Aortic aneurysm and dissection	9	2.19 (1.00-4.16)	12	1.93 (1.00-3.37)	1	0.39 (0.01-2.19)	1	1.24 (0.03-6.93)	23	1.68 (1.07-2.52)
Atherosclerosis	12	4.67 (2.41-8.16)	7	1.88 (0.76-3.88)	1	0.64 (0.02-3.58)	0	NA	20	2.39 (1.46-3.69)
Cerebrovascular diseases	89	2.28 (1.83-2.81)	71	1.18 (0.92-1.49)	39	1.41 (1.00-1.92)	14	1.37 (0.75-2.29)	213	1.56 (1.35-1.78)
Other diseases of arteries, arterioles, capillaries	6	2.18 (0.80-4.75)	9	2.09 (0.96-3.97)	2	1.00 (0.12-3.61)	1	1.38 (0.04-7.70)	18	1.84 (1.09-2.91)
**Infectious diseases**	216	6.53 (5.68-7.46)	153	2.95 (2.50-3.45)	60	2.48 (1.89-3.19)	22	2.54 (1.59-3.85)	451	3.83 (3.48-4.20)
Pneumonia and influenza	79	4.75 (3.76-5.92)	54	2.10 (1.58-2.47)	28	2.32 (1.54-3.35)	11	2.49 (1.24-4.46)	172	2.92 (2.50-3.39)
Syphilis	0	NA	0	NA	0	NA	0	NA	0	NA
Tuberculosis	0	NA	0	NA	1	8.50 (0.22-47.35)	0	NA	1	1.59 (0.04-8.86)
Septicemia	81	8.11 (6.44-10.08)	70	4.37 (3.47-5.52)	21	2.76 (1.71-4.22)	10	3.63 (1.74-6.68)	182	5.01 (4.30-5.79)
Other infectious diseases	56	8.93 (6.74-11.59)	29	2.94 (1.97-4.23)	10	2.28 (1.09-4.20)	1	0.69 (0.02-3.87)	96	4.37 (3.54-5.34)
**Respiratory diseases**	159	3.69 (3.14-4.31)	177	2.50 (2.15-2.90)	74	2.19 (1.72-2.75)	29	2.38 (1.59-3.41)	439	2.75 (2.50-3.02)
Chronic obstructive pulmonary disease and allied Cond	159	3.69 (3.14-4.31)	177	2.50 (2.15-2.90)	74	2.19 (1.72-2.75)	29	2.38 (1.59-3.41)	439	2.75 (2.50-3.02)
**Gastrointestinal diseases**	47	5.23 (3.84-6.96)	25	1.75 (1.13-2.58)	12	2.02 (1.04-3.52)	3	1.60 (0.33-4.676)	87	2.80 (2.24-3.45)
Stomach and duodenal ulcers	2	2.02 (0.24-7.29)	3	2.00 (0.41-5.85)	0	NA	0	NA	5	1.49 (0.48-3.48)
Chronic liver disease and cirrhosis	45	5.63 (4.11-7.54)	22	1.72 (1.08-2.61)	12	2.26 (1.17-3.95)	3	1.81 (0.37-5.29)	82	2.96 (2.35-3.67)
**Renal diseases**	44	3.18 (2.31-4.27)	27	1.21 (0.80-1.77)	14	1.29 (0.71-2.17)	6	1.51 (0.55-3.28)	91	1.79 (1.44-2.20)
Nephritis, nephrotic syndrome and nephrosis	44	3.18 (2.31-4.27)	27	1.21 (0.80-1.77)	14	1.29 (0.71-2.17)	6	1.51 (0.55-3.28)	91	1.79 (1.44-2.20)
**External injuries**	93	3.50 (2.82-4.29)	90	2.13 (1.71-2.61)	34	1.79 (1.24-2.50)	11	1.65 (0.82-2.95)	228	2.41 (2.11-2.75)
Accidents and adverse effects*	43	2.17 (1.57-2.92)	59	1.86 (1.42-2.40)	29	1.98 (1.32-2.84)	10	1.88 (0.90-3.45)	141	1.97 (1.66-2.32)
Suicide and self-inflicted injury	50	8.64 (6.41-11.39)	26	2.81 (1.84-4.12)	5	1.30 (0.42-3.03)	1	0.82 (0.02-4.59)	82	4.08 (3.24-5.06)
Homicide and legal intervention	0	NA	5	3.73 (1.21-8.70)	0	NA	0	NA	5	1.72 (0.56-4.02)
**Other cause of death**	454	3.19 (2.90-3.49)	438	1.88 (1.71-2.06)	165	1.38 (1.18-1.61)	52	1.10 (0.82-1.44)	1109	2.05 (1.93-2.17)
Alzheimers (ICD-9 and 10 only)	10	0.52 (0.25-0.95)	24	0.75 (0.48-1.12)	21	1.18 (0.73-1.81)	8	1.01 (0.44-2.00)	63	0.82 (0.63-1.05)
Diabetes mellitus	39	1.79 (1.27-2.44)	41	1.18 (0.85-1.60)	11	0.70 (0.35-1.24)	4	0.74 (0.20-1.88)	95	1.22 (0.99-1.49)
Congenital anomalies	1	1.60 (0.04-8.91)	1	1.03 (0.03-5.72)	1	2.49 (0.06-13.85)	0	NA	3	1.41 (0.29-4.11)
Certain conditions originating in perinatal period	0	NA	1	233.36 (5.91-1300.22)	0	NA	0	NA	1	108.39 (2.74-603.92)
Complications of pregnancy, childbirth, puerperium	2	536.54 (64.98-1938.17)	0	NA	0	NA	0	NA	2	180.75 (21.89-652.95)
Symptoms, signs and ill-defifined conditions	50	7.10 (5.27-9.36)	34	3.01 (2.08-4.20)	4	0.69 (0.19-1.77)	4	1.86 (0.51-4.77)	92	3.50 (2.82-4.30)
Other	352	3.76 (3.38-4.17)	337	2.19 (1.96-2.43)	128	1.60 (1.34-1.91)	36	1.14 (0.80-1.58)	853	2.37 (2.22-2.54)

*Transport accidents, other external causes of accidental injury and sequelae of transport accidents or other accidents. NA, not applicable.

### Non-cancer causes of death within 1 year following diagnosis

A total of 20335 patients died within 1 year after diagnosis, of which 1769 (8.7%) died because of non-cancer causes. The most common causes of death were CVD (756; 42.7%), followed by other cause of death (454; 25.7%) and infectious diseases (216; 12.2%). Compared with US general populations, EC patients has the higher risk of death from disease of heart (SMR, 3.22; 95% CI, 2.97-3.48), cerebrovascular diseases (SMR, 2.28; 95% CI, 1.83-2.81), pneumonia and influenza (SMR, 4.75; 95% CI, 3.76-5.92), septicemia (SMR, 8.11; 95% CI, 6.44-10.08), COPD (SMR, 3.69; 95% CI, 3.14-4.31), suicide and self-inflicted injury (SMR, 8.64; 95% CI, 6.41-11.39) and diabetes mellitus (SMR, 1.79; 95% CI, 1.27-2.44), along with other cause. In addition, EC patients have a significantly decreased risk of Alzheimer’s disease compare with general populations within 1 year (SMR, 0.52; 95% CI, 0.25–0.95) (**Table 2**).

### Non-cancer causes of death within 1-5 years following diagnosis

A total of 12962 patients died within 1-5 years of diagnosis, of which 1599 (12.3%) died because of non-cancer causes. The most common causes of death were CVD (689; 43.1%), followed by other cause of death (438; 27.4%) and respiratory diseases (177; 11.1%). Compared with the general population, EC patients had higher risk of death from disease of heart (SMR, 1.91; 95% CI, 1.75-2.07), pneumonia and influenza (SMR, 2.10; 95% CI, 1.58-2.47), septicemia (SMR, 4.37; 95% CI, 3.47-5.52), COPD (SMR, 2.50; 95% CI, 2.15-2.90), chronic liver disease and cirrhosis (SMR, 1.72; 95% CI, 1.08-2.61), suicide and self-inflicted injury (SMR, 2.81; 95% CI, 1.84-4.12), along with other cause. Patients’ risk of dying from Alzheimer’s disease was still reduced, but not statistically (SMR,0.75; 95% CI, 0.48-1.12) (**Table 2**).

### Non-cancer causes of death within 5-10 years following diagnosis

A total of 1481 patients died within 5-10 years of diagnosis, of which 645 (43.6%) died because of non-cancer causes. The most common cause of death were CVD (286; 44.3%), followed by other cause of death (165; 25.6%) and COPD (74; 11.5%). Within 5-10 years of diagnosis, patients had a higher risk of death from disease of heart (SMR, 1.72; 95% CI, 1.51-1.96), pneumonia and influenza (SMR, 2.32; 95% CI, 1.54-3.35), septicemia (SMR, 2.76; 95% CI, 1.71-4.22), COPD (SMR, 2.19; 95% CI, 1.72-2.75), chronic liver disease and cirrhosis (SMR, 2.26; 95% CI, 1.17-3.95) and accidents and adverse effects (SMR, 1.98; 95% CI, 1.32-2.84) compared with US general population, along with other causes of death (**Table 2**).

### Non-cancer causes of death after more than 10 years following diagnosis

A total of 395 patients died 10 years after diagnosis, including 235 deaths from non-cancer causes (59.5%) accounted for more than half. The most common causes of death were CVD (112; 47.7%), followed by other cause of death (52; 22.1%) and respiratory diseases (29; 12.3%). After more than 10 years of diagnosis, EC patients showed a higher risk of death from disease of heart (SMR, 1.87; 95% CI, 1.51–2.29), pneumonia and influenza (SMR, 2.49; 95% CI, 1.24–4.46), septicemia (SMR, 3.63; 95% CI, 1.74–6.68), COPD (SMR, 2.38; 95% CI, 1.59-3.41) compared with US general population, along with other causes of death (**Table 2**).

### Risk factor of CVD related death

Given that CVD accounts for a large proportion of non-cancer deaths, we used a multivariate competitive risk analysis to identify predictors associated with CVD related death in EC patients, the results indicated that these following characteristics were independently associated with a higher risk of CVD: 50-70 years old (HR: 2.324; 95% CI: 1.731-3.120), > 70 years old (HR: 4.886; 95% CI: 3.636-6.565), patients who received radiotherapy (HR: 1.180; 95% CI: 1.052-1.324). On the other hand, we found that the following characteristics were independently associated with a lower risk of CVD: female sex (HR: 0.848; 95% CI: 0.755-0.953), poorly differentiated and undifferentiated (HR: 0.843; 95% CI: 0.757-0.938), regional (HR: 0.625; 95% CI: 0.558-0.701) or distant stage (HR: 0.315; 95% CI: 0.271-0.366), married state (HR: 0.883; 95% CI: 0.797-0.978) and diagnosed between 2010-2016 (HR: 0.697; 95% CI: 0.611-0.794) (**Table 3**).

**Table 3:** Multivariate competing risk analysis for predictors of CVD related death in EC patients.

Characteristics		HR	95% CI	P
Age	<50	Ref		
	50-70	2.324	1.731-3.120	< 0.001
	>70	4.886	3.636-6.565	< 0.001
Race	White	Ref		
	Black	1.111	0.959-1.287	0.160
	Other	0.685	0.529-0.888	0.004
Sex	Male	Ref		
	Female	0.848	0.755-0.953	0.006
Grade	I+II	Ref		
	III+IV	0.843	0.757-0.938	0.002
	Unknown	1.135	1.008-1.278	0.037
Stage	Localized	Ref		
	Regional	0.625	0.558-0.701	< 0.001
	Distant	0.315	0.271-0.366	< 0.001
	Unknown	0.779	0.672-0.904	< 0.001
Primary site	Upper esophagus	Ref		
	Mid- esophagus	1.055	0.861-1.292	0.600
	Lower esophagus	1.093	0.903-1.322	0.360
	Overlap	1.091	0.813-1.464	0.560
	Unknown	0.974	0.771-1.230	0.820
Diagnosis year	2000-2009	Ref		
	2010-2016	0.697	0.611-0.794	< 0.001
Marital status	Single	Ref		
	Married	0.883	0.797-0.978	0.018
	Unknown	1.034	0.843-1.268	0.750
Insured status	Uninsured	Ref		
	Medicaid	1.052	0.660-1.677	0.830
	Insured	0.823	0.531-1.275	0.380
	Unknown	1.013	0.651-1.577	0.950
Surgery	No+Unknown	Ref		
	Yes	1.054	0.939-1.184	0.370
Radiation	No+Unknown	Ref		
	Yes	1.180	1.052-1.324	0.005
Chemotherapy	No+Unknown	Ref		
	Yes	0.946	0.843-1.061	0.340

### Effects of radiotherapy on CVD related death with different characteristics

Multivariate competitive risk analysis suggested that radiotherapy was an independent risk factor for CVD related death in EC patients. Therefore, we further analyzed the effects of radiotherapy with different characteristics. we found that the following patients receive radiotherapy has higher risks of CVD related death: 50-70 years old (HR: 1.210; 95% CI: 1.013-1.446), >70 years old (HR: 1.229; 95% CI: 1.054-1.433), white patients (HR: 1.196; 95% CI: 1.053-1.358), male patients (HR: 1.174; 95% CI: 1.030-1.338), regional stage (HR: 1.280; 95% CI: 1.023-1.601), unmarried patients (HR: 1.210; 95% CI: 1.012-1.446), located at the middle third (HR: 1.297; 95% CI: 1.004-1.676) and lower third (HR: 1.234; 95% CI: 1.060-1.437) of the esophagus, specific information see in **Table 4**.

**Table 4:** Effects of radiotherapy on CVD related death in EC patients with different characteristics.

Characteristics		HR	95% CI	P
Age	< 50	0.660	0.301-1.448	0.300
	50-70	1.210	1.013-1.446	0.035
	> 70	1.229	1.054-1.433	0.009
Race	White	1.196	1.053-1.358	0.006
	Black	1.110	0.810-1.519	0.520
Sex	Male	1.174	1.030-1.338	0.016
	Female	1.193	0.936-1.520	0.150
Stage	Localized	1.161	0.930-1.448	0.190
	Regional	1.280	1.023-1.601	0.031
	Distant	1.091	0.855-1.393	0.480
Grade	I+II	1.180	0.979-1.422	0.082
	III+IV	1.213	0.999-1.473	0.051
Marital status	Married	1.142	0.974-1.338	0.100
	Single	1.210	1.012-1.446	0.036
Primary site	Upper esophagus	0.990	0.598-1.64	0.970
	Mid- esophagus	1.297	1.004-1.676	0.046
	Lower esophagus	1.234	1.060-1.437	0.007

### Effects of comprehensive treatment measures on CVD related death in EC patients

Because some patients in our cohort received combination therapies, we further divided patient treatment measures to determine the effects of different treatments on CVD related death in EC patients through multivariate competitive risk analysis, the results indicated that these following characteristics were independently associated with a higher risk of CVD: 50-70 years old (HR: 2.307; 95% CI: 1.719-3.097), > 70 years old (HR: 4.949; 95% CI: 3.608-6.516). On the other hand, we found that the following characteristics were independently associated with a lower risk of CVD: female sex (HR: 0.848; 95% CI: 0.755-0.953), poorly differentiated and undifferentiated (HR: 0.848; 95% CI: 0.762-0.944), regional (HR: 0.635; 95% CI: 0.565-0.713) or distant stage (HR: 0.333; 95% CI: 0.286-0.388), married (HR: 0.883; 95% CI: 0.797-0.979), diagnosed between 2010-2016 (HR: 0.696; 95% CI: 0.610-0.793) and patients received chemotherapy alone compared with patients without any treatment measures (HR: 0.702; 95% CI: 0.551-0.895) (**Table 5**).

**Table 5:** Effects of comprehensive treatment measures on CVD related death in EC patients.

Characteristics		HR	95% CI	P
Age	< 50	Ref		
	50-70	2.307	1.719-3.097	< 0.001
	> 70	4.949	3.608-6.516	< 0.001
Race	White	Ref		
	Black	1.115	0.963-1.292	0.150
	Other	0.686	0.529-0.890	0.005
Sex	Male	Ref		
	Female	0.848	0.755-0.953	0.006
Grade	I+II	Ref		
	III+IV	0.848	0.762-0.944	0.003
	Unknown	1.134	1.007-1.276	0.038
Stage	Localized	Ref		
	Regional	0.635	0.565-0.713	< 0.001
	Distant	0.333	0.286-0.388	< 0.001
	Unknown	0.788	0.677-0.918	0.002
Primary site	Upper esophagus	Ref		
	Mid- esophagus	1.060	0.865-1.298	0.580
	Lower esophagus	1.102	0.910-1.334	0.320
	Overlap	1.099	0.819-1.474	0.530
	Unknown	0.980	0.775-1.238	0.860
Diagnosis year	2000-2010	Ref		
	2010-2016	0.696	0.610-0.793	< 0.001
Marital status	Single	Ref		
	Married	0.883	0.797-0.979	0.018
	Unknown	1.030	0.840-1.264	0.770
Insured status	Uninsured	Ref		
	Medicaid	1.046	0.656-1.666	0.850
	Insured	0.822	0.530-1.273	0.380
	Unknown	1.006	0.646-1.566	0.980
Treatment measures	No+Unknown	Ref		
	Surgery only	1.075	0.905-1.276	0.410
	Radiotherapy only	0.955	0.788-1.156	0.630
	Chemotherapy only	0.702	0.551-0.895	0.004
	Surgery+ Radiotherapy	1.300	0.809-2.087	0.280
	Surgery+ Chemotherapy	0.809	0.532-1.231	0.320
	Radiotherapy+ Chemotherapy	1.133	0.988-1.300	0.074
	Surgery+ Radiotherapy+ Chemotherapy	1.084	0.899-1.306	0.400

## Discussion

Analysis of the mortality rate and the main causes of death of malignant tumors are helpful to master the disease characteristics of malignant tumors, and is of great significance to the control and management of tumors. According to a study about the causes of death among cancer patients in China, EC is the fourth leading cause of in-hospital deaths among cancer patients (16). With the spread of neoadjuvant therapy, the current median survival time for EC patients has improved significantly (4). With the increasing number of long-term EC survivors, non-cancer causes have become one of the main causes of death in EC patients, especially CVD related death, which are currently the second leading cause of death, after cancer itself (10), and the trend is still rising. As the diversification of combination therapies leads to long-term survival in more cancer patients, it is increasingly important to identify non-cancer deaths due to comorbidities or side effects due to treatment toxicity.

Recent years, several studies have suggested that cancer patients have a higher risk of CVD related death compared with general population (12, 14). Wang’s study showed that the cardiovascular mortality rates of 1, 3 and 5 years in cancer patients were 6.0%, 10.8% and 17.9%, respectively, and the total cardiovascular mortality rates were 25.8% (17). According to previous report, cancer patients have a higher risk of thromboembolism compare with general population, especially in lung and pancreatic cancer (18). In our research, EC patients have a higher risk of death from CVD than the general population during the whole follow-up periods (SMR, 2.11; 95% CI, 2.01–2.20), the highest risk of death from CVD occurred within 1 year after diagnosis (SMR, 3.04; 95% CI, 2.83–3.27). At the beginning of cancer diagnosis, patients are often at increased risk of CVD due to psychological stress such as anxiety and stress, these emotional states may be increased pressure on the heart due to the increased burden of disease, leading to death from cardiovascular causes, this phenomenon were particularly pronounced in esophageal, liver, pancreatic and lung cancers (19). In addition, EC patients are at risk for long-term cardiac sequelae due to radiotherapy, chemotherapy or surgery during treatment, which may also contribute to the high incidence of CVD related death (20).

CVD are the most common cause of non-cancer death in EC patients, it is significant to select high-risk EC patients of CVD related death and develop appropriate measures to prevent potential risk of it. Previous studies indicated that male sex, black race, advanced age, receive radiotherapy were associated with a significantly increased risk of death from CVD (17, 20). Male cancer patients have a greater risk of death from CVD compared to women (17), our research came to the same conclusion, this phenomenon may be due to hormonal differences (21). Another possible reason were that men tend to have worse lifestyle habits, such as drinking and smoking, which have been reported to be independent risk factors for CVD (22–24). According to previous studies, black are more likely to died from CVD than other races (25, 26), this may be due to the higher risk of venous thromboembolism in black cancer patients (26). Our research showed that black patients are at greater risk of CVD related death, but there was no statistical significance (HR:1.111; P=0.160), this may be due to the small percentage of blacks in the overall study cohort (11.3%), so may be it doesn’t reflect actual CVD related death in the whole black population, therefore, further investigation about this issue in EC patients is still necessary. In our research, older patients are more likely to died from CVD, which is in line with previous study (17). The reason may be that elderly patients has a higher incidence of comorbidities, which may result higher risk of cardiovascular events, but the specific mechanism still needs further analysis. In addition, multivariate competitive risk analysis suggested that EC patients with poorly and undifferentiated (HR: 0.843; 95% CI: 0.757-0.938) and distant tumor stage (HR: 0.315; 95% CI: 0.271-0.366) has lower risk of CVD related death, low degree of differentiation and distant stage are adverse prognostic factors of EC patients (27, 28), since the proportion of CVD related death increased with the increase in years of diagnosis, leading to the possibility that these patients may not have a long enough life expectancy to died from CVD. Married patients showed a lower tendency to died from CVD than unmarried patients (HR: 0.883; 95% CI: 0.797-0.978), they often receive more physical and emotional care, encouragement and support than unmarried patients (29), that could be one of the reason. Our study revealed that EC patients diagnosed between 2010-2016 had a lower risk of CVD related death. With the advances in imaging and laboratory medicine, such as cardiac magnetic resonance imaging and echocardiography, as well as the widespread clinical use of troponin and n-terminal b-type natriuretic peptide, resulting more and more early detection and intervention of heart disease (30–32), in addition, the comprehensive treatment measures of EC continues to improve (4–8), these advances in medical technology may have significantly reduced heart-specific mortality in recent years.

Many studies established that cancer patients without surgery may lead to a significantly increased risk of CVD related death (17, 20, 33), but there have also been studies showing that surgery can increase the risk of venous thromboembolism (34, 35). Our study indicated that surgical treatment is an risk factor for CVD related death but there was no statistical significance (HR: 1.054; P=0.370). Khorana proposed that thromboembolism is the leading cause of death in cancer patients who received chemotherapy (10), in our research, patients who received chemotherapy alone had a lower risk of CVD related death than patients who did not received any treatments (HR: 0.702; P=0.004). It has been claimed that anticoagulant therapy can prolong the survival time of cancer patients, low molecular weight heparin and warfarin are more and more commonly used in cancer patients during chemotherapy in recent years, which to some extent reduces the probability of thromboembolic events in cancer patients (18). In addition, patients received regularly chemotherapy can have better health monitoring during treatment and receive more timely intervention when at risk for thromboembolism to avoid subsequent adverse events. These reasons may explain the lower risk of CVD related death in EC patients who receiving chemotherapy alone.

Advances in cancer treatment, while significantly reducing mortality from primary cancer, may increase the risk of death from causes other than primary cancer, such as non-cancer comorbidities. Radiotherapy can bring survival benefits to EC patients (4), but the short and long-term side effects of radiotherapy cannot be ignored. Our study indicated that EC patients who received radiotherapy had a higher risk of death from CVD (HR:1.180; P=0.005). This is consistent with the conclusions of previous analysis (20). Long-term cardiotoxicity caused by radiotherapy has been previously reported, mainly including myocardial fibrosis, pericardial disease, coronary artery disease, valvular disease and arrhythmias (20). It has been reported that abnormal increase of myocardial perfusion, decrease of ejection fraction and pericardial effusion occurred after chemoradiotherapy by imaging examination (36–38). In addition, given that the heart is anatomically located close to the esophagus, it is expected that the radiation process will affect the heart, these above side effects may lead to cardiovascular system injury, but the exact mechanism is still unclear and further research is still needed. Therefore, despite the survival benefits of radiotherapy for EC patients, its potential cardiac toxicity and side effects should not be ignored, radiation-induced cardiotoxicity should be considered in long-term follow-up management of EC patients.

Our study have several limitations: first, factors such as patient’s comorbidities (hyperlipidemia, diabetes, dyslipidemia, obesity and history of CVDs), bad habits (alcohol use and smoking habit), family history, detailed information of radiation and chemotherapy (type, dosage, and duration) may influence CVD related death in EC patients, but these information are not included in the SEER database (12, 14). Second, because only patients diagnosed between 2000 and 2016 were included, some patients diagnosed late do not have adequate follow-up time. Finally, this study was retrospective analysis, which may be biased in data selection.

## Conclusion

Non-cancer deaths account for a significant proportion of death in EC patients, the highest numbers of non-cancer deaths were caused by CVD. Patients, who were 50-70 years old, >70 years old, male sex, well differentiated and moderately differentiated, localized stage, unmarried state, diagnosed between 2000-2009 and receive radiotherapy had a significantly higher risk of death from CVD. Compared with EC patients without any treatments, patients received chemotherapy alone has lower risk of CVD related death. Regular CVD screening and CVD risk factors control are of great significance for the follow-up of EC patients.

## Data availability statement

Publicly available datasets were analyzed in this study. This data can be found here: https://seer.cancer.gov/.

## Author contributions

YX and LF designed the research. YX, ML, JH, LF performed the study and analyzed the data. YX and ML wrote the paper and interpreted the data. LF, ML and JH help to revised manuscript. All authors read and approved the final manuscript. All authors contributed to the article and approved the submitted version.

## Funding

This work was supported by the Changzhou No.2 People’s Hospital Fund (2019HZD001).

## Acknowledgments

The authors acknowledge contributions from SEER program.

## Conflict of interest

The authors declare that the research was conducted in the absence of any commercial or financial relationships that could be construed as a potential conflict of interest.

## Publisher’s note

All claims expressed in this article are solely those of the authors and do not necessarily represent those of their affiliated organizations, or those of the publisher, the editors and the reviewers. Any product that may be evaluated in this article, or claim that may be made by its manufacturer, is not guaranteed or endorsed by the publisher.
